# Impact of cardiac patch alignment on restoring post-infarct ventricular function

**DOI:** 10.1007/s10237-024-01877-9

**Published:** 2024-08-01

**Authors:** Koen L. P. M. Janssens, Peter H. M. Bovendeerd

**Affiliations:** Department of Biomedical Engineering, Eindhoven University of Technology, 5600MB Eindhoven, The Netherlands

**Keywords:** Cardiac patch, Computational modeling, Myocardial infarction, Tissue engineering

## Abstract

Acute myocardial infarction (MI) leads to a loss of cardiac function which, following adverse ventricular remodeling (AVR), can ultimately result in heart failure. Tissue-engineered contractile patches placed over the infarct offer potential for restoring cardiac function and reducing AVR. In this computational study, we investigate how improvement of pump function depends on the orientation of the cardiac patch and the fibers therein relative to the left ventricle (LV). Additionally, we examine how model outcome depends on the choice of material properties for healthy and infarct tissue. In a finite element model of LV mechanics, an infarction was induced by eliminating active stress generation and increasing passive tissue stiffness in a region comprising 15% of the LV wall volume. The cardiac patch was modeled as a rectangular piece of healthy myocardium with a volume of 25% of the infarcted tissue. The orientation of the patch was varied from 0 to $150^ \circ$ relative to the circumferential plane. The infarct reduced stroke work by 34% compared to the healthy heart. Optimal patch support was achieved when the patch was oriented parallel to the subepicardial fiber direction, restoring 9% of lost functionality. Typically, about one-third of the total recovery was attributed to the patch, while the remainder resulted from restored functionality in native myocardium adjacent to the infarct. The patch contributes to cardiac function through two mechanisms. A contribution of tissue in the patch and an increased contribution of native tissue, due to favorable changes in mechanical boundary conditions.

## Introduction

Myocardial infarction (MI) is one of the leading causes of death worldwide, affecting almost 10% of all individuals aged over 60 (Salari et al. 2023). When the heart survives the acute phase of MI, a remodeling process starts (Sutton and Sharpe 2000). Over time, the injured myocardium is gradually replaced by relatively stiff scar tissue (Gupta et al. 1994; McGarvey et al. 2015; Fomovsky and Holmes 2010). The loss of cardiomyocytes and fibrosis are important contributors to left ventricular dysfunction and play an important role in LV remodeling (Burchfield et al. 2013). The functional consequences include for example prolonged isovolumic relaxation and increased diastolic stiffness (Thune and Solomon 2006). Eventually, this may lead to heart failure (HF) (Frantz et al. 2022; French and Kramer 2007).

Though heart transplantation remains the preferred treatment for end-stage heart disease in adults, the scarcity of donor hearts has stimulated the use of left ventricular assist devices (LVAD) as destination therapy for individuals ineligible for transplantation (Kirklin et al. 2014). While, LVADs contribute to improved survivability and long-term outcomes in such patients, they are associated with several significant complications. Some of these complications are a result of the compromised health of end-stage heart failure patients, while others are device-related, including bleeding events, infection, device thrombosis, stroke and pump malfunction (Kilic et al. 2015). As a result, LVAD treatment remains associated with elevated mortality and complication rates post-implantation (Jezovnik et al. 2017; Dunlay et al. 2016).

Novel approaches are being developed with the aim of providing a long-lasting solution for this patient group by, for example, mitigating the effects of fibrosis or creating new constructs which directly improve cardiac function (Morfino et al. 2023). Such constructs include cardiac patches that are disposed over the infarct region and can be seeded with cardiomyocytes. These patches may actively contribute to cardiac function through the contractile properties of the cells but also affect the function of tissue adjacent to the infarct. Indeed, in animal models of acute MI, it was found that a contractile cardiac patch can reduce adverse ventricular remodeling and increase cardiac function in remote tissue and the border zone (Gao et al. 2018; Wendel et al. 2014; Montgomery et al. 2017). However, in models of chronic MI, no improvements in cardiac function were found (Riegler et al. 2015; von Bibra et al. 2022; Kawamura et al. 2012). Since, these studies differ from each other in many aspects, such as animal model, patch size, cell source or infarct characteristics, drawing conclusions is difficult. In addition, results are generally quantified in terms of cardiac function at organ level. However, to understand the mechanisms underlying the changes in pump function, information on function at tissue level, such as stress and strain, is crucial.

Computational models can assist in the interpretation of experimental studies since they provide information, for example on tissue level mechanics that cannot be acquired experimentally. They can also be used to identify the role of certain design elements in overall patch functionality.

In this study, we aim to evaluate the effect of one of these choices in patch design: the orientation of the fibers in the patch with respect to fiber orientation in the native heart. We focus on fiber orientation since our model of the infarcted left ventricle showed that infarct-induced effects on stress and strain patterns in the tissue adjacent to the infarct depend strongly on the local orientation of the myofibers with respect to the infarct (Janssens et al. 2023). In the present study, the latter model was extended with a contractile patch. We varied the orientation of the patch and analyzed to which extent the enhancement of pump function stems from the contribution of the patch tissue that is added, and to which extent it is due to changes in work in the native, still healthy tissue.

## Methods

### Model of cardiac mechanics

The finite element model of cardiac mechanics from (Bovendeerd et al. 2009) was employed with the extension as proposed in (Janssens et al. 2023) to include the effects of MI. The model was further extended to include a cardiac patch fixed over the infarct site. The constitutive equations, boundary conditions, closed-loop lumped parameter model and parameter values used in this study are verbatim to those in the original. A brief description of this model is provided here. A complete overview was added in Appendix A.

**Fig. 1:**
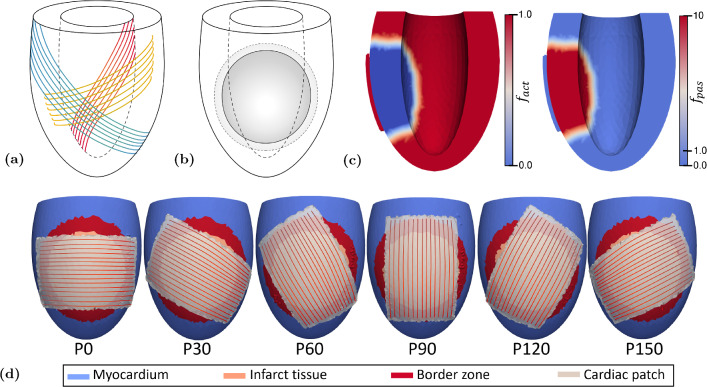
**(a)** Schematic of LV geometry with fiber orientation depicted near the epicardium (blue), midwall (yellow) and endocardium (red). **(b)** Region of infarcted tissue and the border zone depicted in gray. **(c)** Section of LV geometry with factors $f_\text{act}$ (left) and $f_\text{pas}$ (right) displayed in color. **(d)** The different cardiac patch orientations with an angle of $0^{\circ }$ (left) to $150^{\circ }$ (right) with respect to the circumferential plane

In short, the model employs an ellipsoidal geometry (see Figure 1a). The myocardium is modeled as a nonlinearly elastic, transversely isotropic nearly-incompressible material with an active stress component that acts parallel to the fiber direction. The Cauchy stress tensor is given by:

1 $$\begin{aligned} \begin{aligned} \varvec{\sigma } = f_\text{ pas } \varvec{\sigma }_\text{ pas } + f_\text{ act } \sigma _\text{ act }[\varvec{\vec {e}}_f \varvec{\vec {e}}_f + \beta (\varvec{\vec {e}}_s \varvec{\vec {e}}_s + \varvec{\vec {e}}_n \varvec{\vec {e}}_n)] \end{aligned} \end{aligned}$$

Here, $\varvec{\vec {e}}_f, \varvec{\vec {e}}_s, \varvec{\vec {e}}_n$ represent the unit vectors in fiber, sheet and sheet-normal direction, respectively. $\varvec{\sigma }_\text{pas}$ and $\sigma _\text{act}$ represent the passive and active components of the total stress tensor, respectively. Factors $f_{pas}$ and $f_{act}$ allow for the variation of material properties within the myocardium. Parameter $\beta$ describes the level of active stress development in the cross-fiber direction. A rule-based method was used to define the fiber orientation in terms of a helix angle $\alpha _h$ and transverse angle $\alpha _t$. These represent the angle between the circumferential direction and the projection of the fiber vector on the circumferential-longitudinal and circumferential-transmural plane, respectively. The helix angle ranged from $+70^ \circ$ at the endocardial surface, through $+20^\circ$ at midwall until $-45^\circ$ at the epicardial surface (see Figure 1a). Development of $\sigma _\text{act}$ was initiated simultaneously throughout the LV wall with a cycle time of 800 ms. The healthy heart was simulated by prescribing a value of 1 for both $f_\text{pas}$ and $f_\text{act}$ throughout the LV to serve as a reference, labeled H. Material parameter settings are shown in Table 1 in Appendix  A.

The region of infarcted tissue was modeled to be representative of that resulting from an occlusion of the circumflex artery, as a circle in the circumferential-longitudinal plane of the geometry. The circle had a radius of 2 cm and its midpoint was located roughly 1.5 cm below the LV equator (see Figure 1b). We assumed a case of chronic MI with a ten-fold increase in passive tissue stiffness and no active stress generation. Therefore, factors $f_\text{pas}$ and $f_\text{act}$ were set to ten and zero, respectively within the infarct region. The value for $f_\text{pas}$ was chosen based on the stress–strain relations from chronic infarctions in McGarvey et al. (2015) and Gupta et al. (1994). A border zone, separating healthy from infarcted myocardium, was included as a gradual, linear transition in material properties over a width of 7.5 mm (Lee et al. 1981; Sakai et al. 1985). The infarct region and the border zone each have a size of about 14 ml, equivalent to 10% of the total wall volume. In view of the complete loss of contractility in the core infarct, and the average loss of 50% of contractility in the border zone, we quantify this infarct as comprising 15% of the total LV wall volume. We refer to this simulation of chronic myocardial infarction as CMI.

### Modeling the cardiac patch

The cardiac patch was modeled as a rectangular strip of contractile tissue measuring 6 cm in length and 4 cm in width with a thickness of 2 mm. Consequently, it has a volume of about 5 ml. The patch was centered on top of the infarct region and fixed to the epicardial surface. Fibers were aligned along the long side of the patch and activated simultaneously with the LV. We assumed material properties within the cardiac patch equal to those of healthy myocardium and set factors $f_\text{pas}$ and $f_\text{act}$ to a value of 1 (see Figure 3c). Six different patch orientations were modeled, where the long side was oriented at an angle with respect to the LV circumferential direction. This angle was varied from 0 to 150 degrees with steps of $30^\circ$ (see Figure 1d). These models were labeled P0 to P150.

### Parameter variations

The results from models H, CMI and P0 through P150 depend on the settings of the model parameters, in particular on the settings of the material parameters. The influence of these settings was evaluated in additional simulations.

Firstly, studies characterizing the material properties of infarct tissue indicate that anisotropy changes over time during the remodeling process (McGarvey et al. 2015). We evaluated the effect of the degree of anisotropy in simulation sets ANISO- and ANISO+. In ANISO-, we decreased parameter a3 to zero, thereby making the tissue isotropic. In ANISO+, we increased parameter a3 to 6, thereby increasing tissue anisotropy. Changes in anisotropy affect the passive pressure-volume relation, the end-diastolic volume, and consequently also the systolic behavior. To isolate the effect of anisotropy from these effects, we matched the same end-diastolic volume of the infarct models by slightly increasing parameter $f_\text{pas}$ from 10 to 11.25 in simulation ANISO-, and slightly decreasing it to 8.75 in simulation ANISO+. Per set we simulated the infarct case CMI, and the patch orientation cases P30 and P120.

Secondly, we assumed active stress to act along the fiber direction only, by setting $\beta$=0 in equation 1. However, experiments suggest that an active stress component might act in cross-fiber direction as well (Lin and Yin 1998). To evaluate the effect of cross-fiber active stress the parameter beta was set to 0.25 in simulation set CF. In this set we simulated the healthy case H, the infarct case CMI, and the patch orientation cases P30 and P120.

### Simulations and postprocessing

The model was implemented in the FEniCS open-source computing platform and benchmarked against the preceding SEPRAN implementation, used in Bovendeerd et al. (2009) (chapter 2 of (Barbarotta 2020)). FEniCS’ built-in biconjugate gradient stabilized method was used in the linear solver. Absolute residual tolerance was set to 1e-4, calculated as the L2 norm. A timestep of 2 ms was used. The mesh was divided into two sections corresponding to the LV and cardiac patch with elements conforming to the ellipsoidal surface boundary of the LV. Subsequently, all elements located in the border zone and cardiac patch were refined using the FEniCS edge bisection algorithm. Average element size was equal to 4.8 $\mu$l in the healthy myocardium and core infarct region and equal to 0.6 $\mu$l in the patch and border zone. The analytically defined borders between the healthy tissue, the border zone, and the core infarct were thus approximated within 0.9 mm. The resulting mesh consisted of 69,299 quadratic tetrahedral elements with 302,493 degrees of freedom. Factors $f_\text{act}$ and $f_\text{pas}$ were defined in the integration points of each element such that a smooth transition between core infarct, border zone and healthy myocardium was ensured but also a sharp transition between the LV and cardiac patch (see Figure 1c).

For every simulation, five cardiac cycles were computed with a heart rate of 75 bpm such that a hemodynamic steady state was achieved with a change in stroke volume of less than 1% between consecutive cycles. Global cardiac function was assessed using the pressure-volume signals of the last cardiac cycle and stroke work $W_\text{hemo}$ was computed according to:

2 $$\begin{aligned} W_\text{hemo} = \int _\text{cycle} p_\text{lv} \; \text {d}V \end{aligned}$$

The total amount of mechanical work, performed by the LV and cardiac patch over the cardiac cycle, was computed according to:

3 $$\begin{aligned} \begin{aligned} W_{\text{mech, i}} =&\int _{Vi} \int _\text{cycle} \! \sigma _\text{act} \; \text {d}\varepsilon \; \text {d}V_i \\& \text {where}\quad \varepsilon = \ln \left( \frac{l_\text{s}}{l_{\text{s},0}}\right) , \; i = [\text {lv, patch}] \end{aligned} \end{aligned}$$

where $l_\text{s}$ and $l_\text{s,0}$ represent the actual and reference sarcomere length, respectively and $\sigma _\text{act}$ the active component of the Cauchy stress tensor in fiber direction. For all patch simulations, $\Delta W_{\text{mech, i}}$ was reported as the change in $W_{\text{mech, i}}$ with respect to case CMI. Local tissue function was evaluated using Cauchy stress and logarithmic strain in fiber direction over the course of the last cardiac cycle in 12 sectors adjacent to the border zone (see Figure 3a). Within each sector, representative fiber stress–strain loops were created as the average of the loops in ten locations. For the parameter variation sets ANISO-, ANISO+ and CF, the 12 sectors were condensed into four by averaging over sectors 12, 1 and 2, sectors 3, 4 and 5, sectors 6, 7 and 8 and sectors 9, 10 and 11.

## Results

### Hemodynamics

**Fig. 2:**
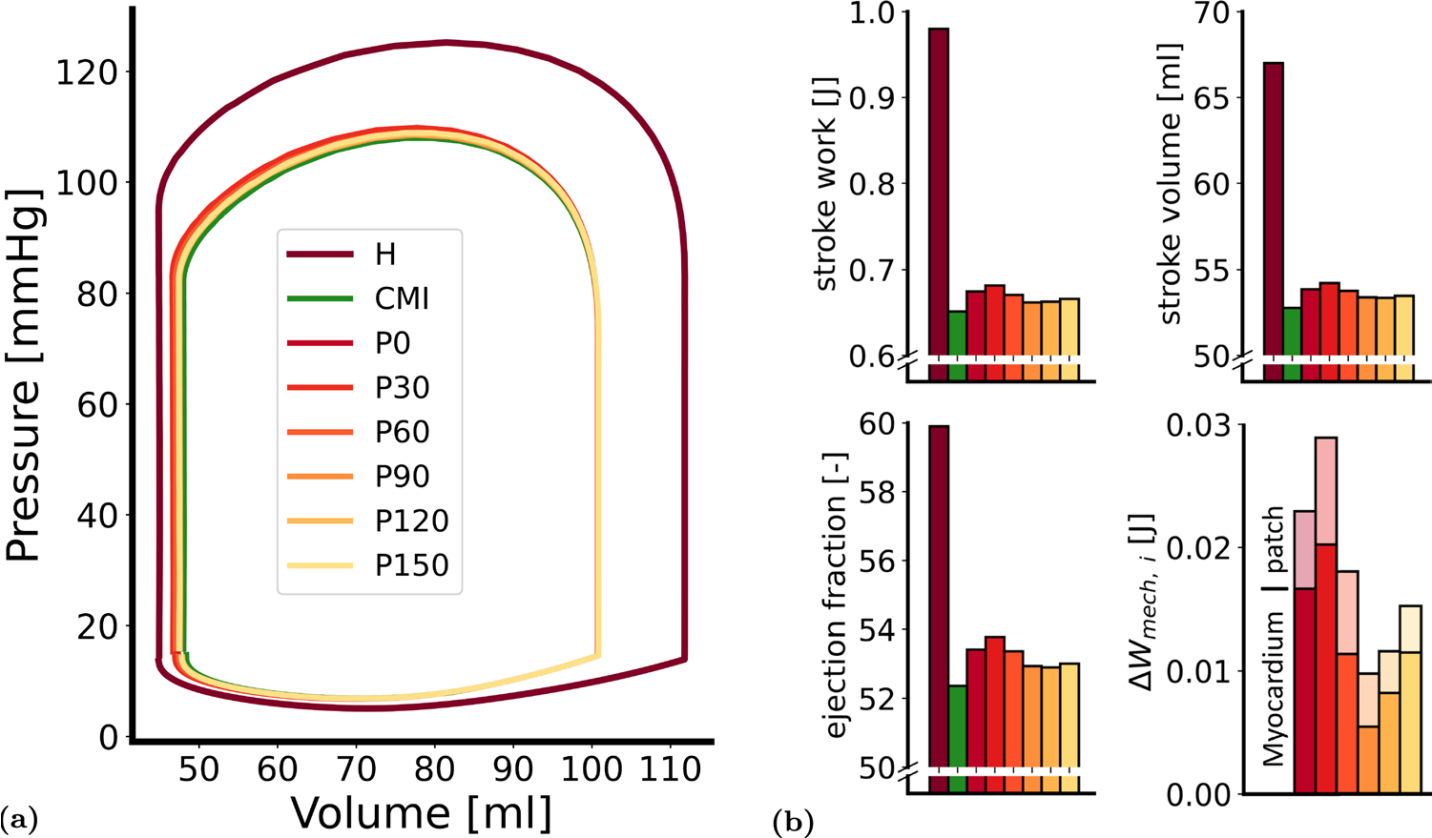
Results from the REF simulation set: (**a**) LV pressure-volume loops and (**b**) cardiac function quantified in stroke work ($W_\text{hemo}$), stroke volume (SV), ejection fraction (EF) and mechanical work following patch implantation ($\Delta W_{\text{mech,} \text{i}}$)

Figure 2 shows pressure-volume loops and bar charts for stroke volume (SV), ejection fraction (EF), stroke work ($W_\text{hemo}$) and work increase due to patch implantation ($\Delta W_{\text{mech, i}}$) of all simulations. Hemodynamics of the healthy heart were representative of the healthy adult heart. Cardiac function was quantified by a SV of 67.0 ml, EF of 59.9% and $W_\text{hemo}$ of 0.98 J. In chronic MI (CMI), SV, EF and $W_\text{hemo}$ decreased by 14.3 ml, 7.7% and 0.33 J, respectively.

In all cardiac patch simulations, $p_{\text{lv}, \text{max}}$ increased, end-systolic volume decreased and end-diastolic volume remained virtually unaffected, with changes below 0.03 ml compared to the CMI case. The extent to which cardiac function was restored varied with patch orientation. This recovery was largest in P30, where SV, EF and $W_\text{hemo}$ increased by 1.5 ml, 1.5% and 0.03 J, respectively. It was smallest in P90 where these increases were reduced to 0.6 ml, 0.5% and 0.01 J, respectively. The increase in $W_{\text{mech,} \text{i}}$ following patch implantation can be attributed to the work in the cardiac patch ($\Delta W_{\text{mech, patch}}$) and increase in work in the LV ($\Delta W_{\text{mech, lv}}$). The relative contribution of the patch $\Delta W_{\text{mech, patch}}$ was highest in P90 (44%), and lowest in P150 (24%), and equal to 30% in the optimal case P30.

### Local tissue function

**Fig. 3:**
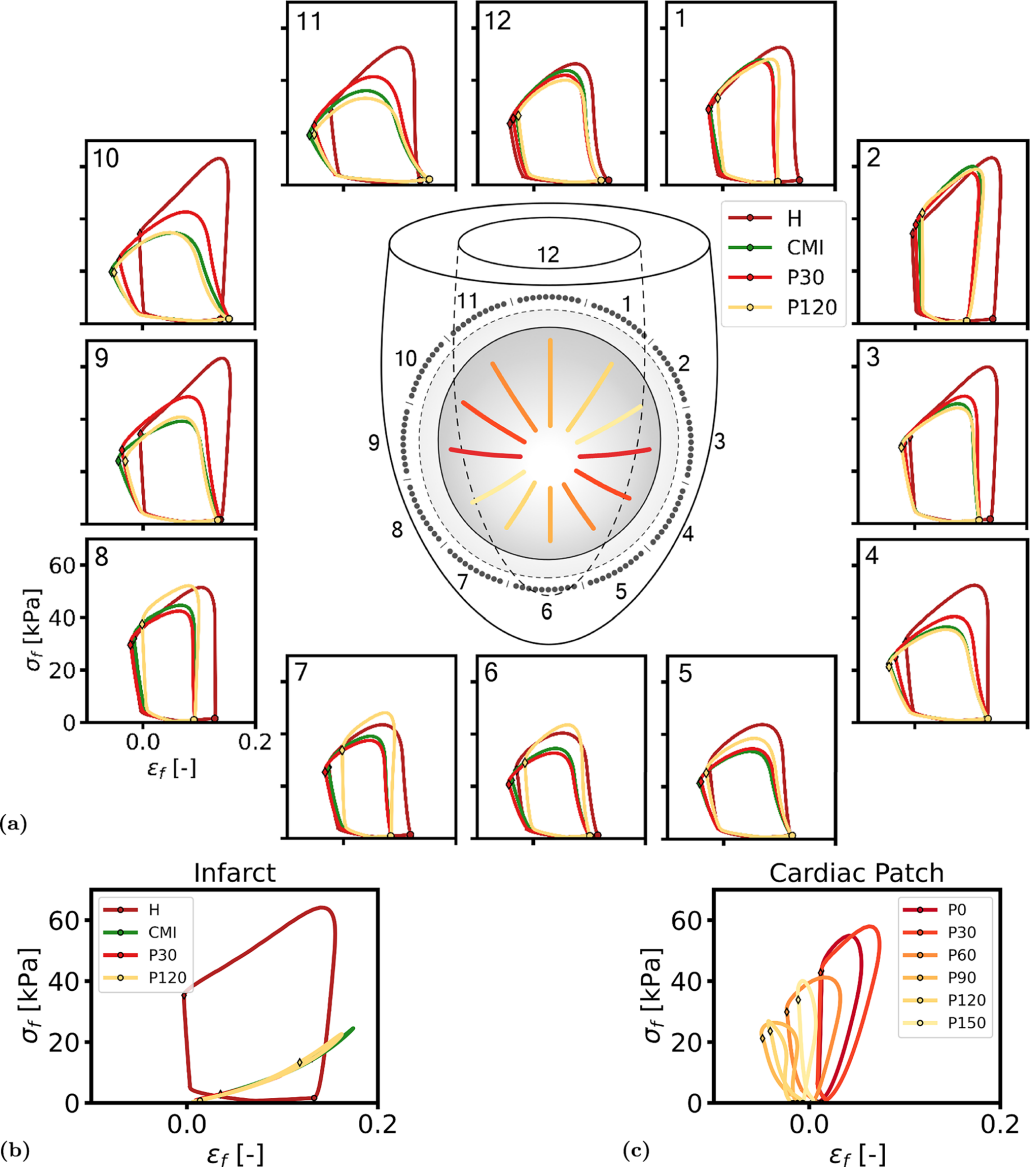
Results from the REF simulation set: (**a**) fiber stress vs fiber strain plots, averaged in 12 sectors within tissue neighboring the infarction and border zone for simulation set REF. (**b**) averaged stress–strain plots in the core infarct region for simulations H, CMI, P30 and P120. (**c**) stress–strain plots averaged in the cardiac patch for simulations P0 through P150. The orientation of the cardiac patch has been shown in a colored line corresponding to the color in the stress–strain loops

Figure 3a shows fiber stress vs. strain loops over the cardiac cycle for the 12 sectors neighboring the infarct region. In the healthy heart (H), the stress–strain loops follow a similar pattern in all sectors. Fiber stress and strain increase to 1.6 kPa and 0.16, respectively towards the end of diastole (denoted by $\circ$). During isovolumic contraction, fiber strain remains about constant, while fiber stress increases to a value between 48 and 61 kPa. Over the course of ejection, both fiber stress and strain decrease before reaching the end of systole (denoted by $\lozenge$). During isovolumic relaxation, fiber strain remains about constant, while stress decreases.

In the chronically infarcted heart (CMI), sectors 3, 4, 5, 9, 10 and 11 show stress–strain loops that are skewed more to the left (see Figure 3a). Compared to H, they show similar strain in diastole but shorten during isovolumic contraction and have lower end-systolic strain. Peak fiber stress was also reduced by 8–30 kPa depending on the sector. Fibers in sectors 1, 2, 6, 7, 8 and 12 show a decrease in end-diastolic strain between 0.01 and 0.04, while end-systolic strain is equal to that in the H case. Peak fiber stress is reduced by up to 8 kPa. Figure 3b shows the average stress–strain loops in the infarcted area. Compared to H, the surface area enclosed by the stress–strain loop in CMI disappeared.

Cardiac patch implantation induced changes in local tissue function. For clarity, in Figure 3a we show stress–strain loops for two patch simulations only: P30, yielding the largest improvement in cardiac function, and P120, where the patch is oriented perpendicular to that in P30. Simulation P30 showed increased fiber stress and more upright stress–strain loops in sectors 3, 4, 9, 10 and 11. Stress–strain loops in the remaining sectors were similar to those found in CMI. In simulation P120, increased peak fiber stress and more upright stress–strain loops were found in sectors 5–8, while loops in the remaining sectors were similar to CMI. The remaining simulations P0, P60, P90 and P150 showed similar changes in stress–strain loops compared to CMI with a gradual transition between sectors.

In the infarcted area, stress–strain loops remain closed (see Figure 3b). The maximum strain excursion decreased depending on the orientation of the cardiac patch, while the slope of the passive stress–strain relation was hardly affected. The stress–strain loops in the cardiac patch show a large variation in size and shape (see Figure 3c). In simulation P30, fiber strain remains constant during diastole and increases to 0.07 during the first part of contraction. Peak fiber stress reaches about 55 kPa. In simulation P120, fiber strain decreases throughout systole to $-$0.06 at end-systole. Peak fiber stress was equal to 26 kPa.

### Parameter variations

**Fig. 4:**
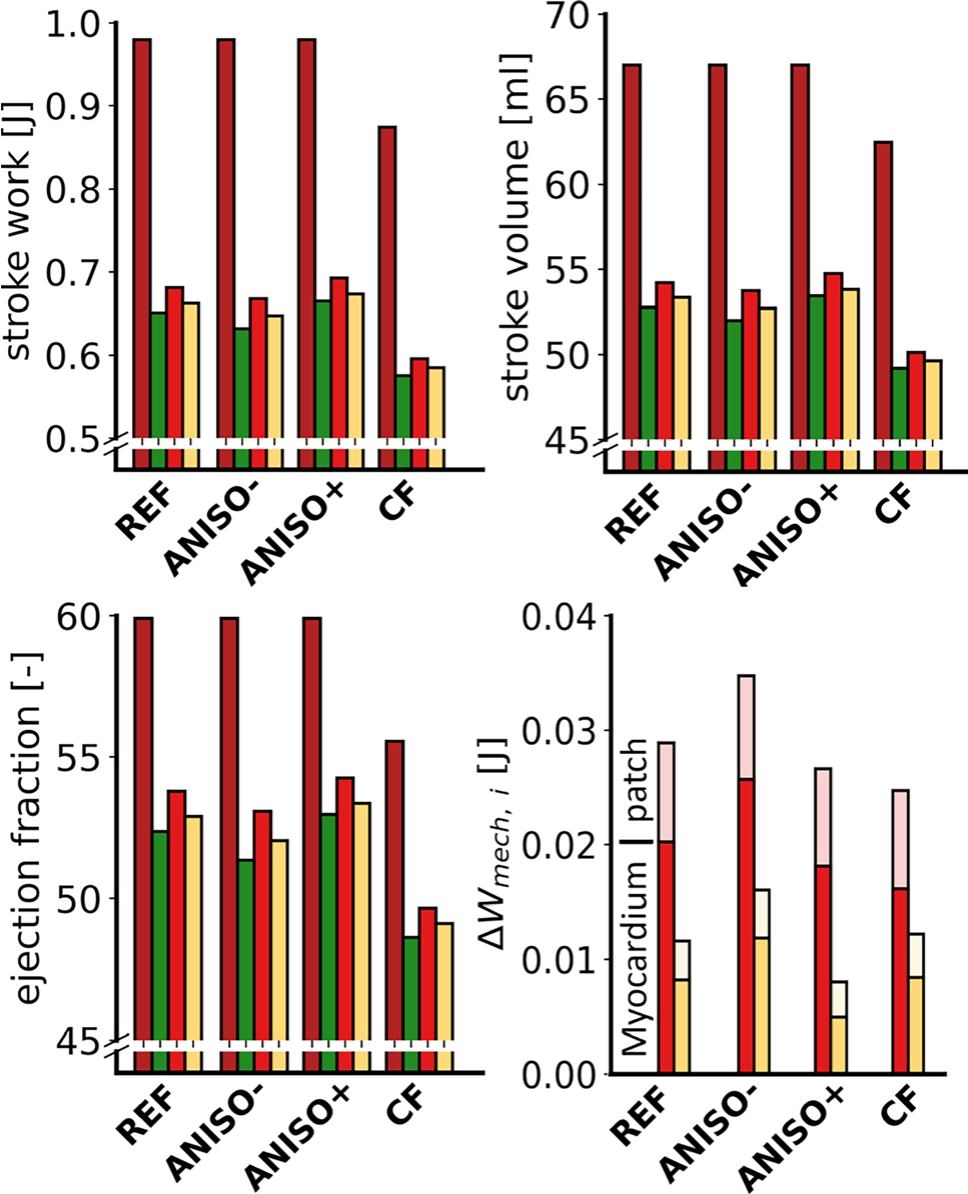
Results for the H, CMI, P30 and P120 simulations for sets REF, ANISO-, ANISO+ and CF. Cardiac function is quantified in stroke work ($W_\text{hemo}$), stroke volume (SV), ejection fraction (EF) and mechanical work following patch implantation ($\Delta W_{\text{mech,} \text{i}}$)

Figure 4 shows the hemodynamic markers for sets ANISO-, ANISO+ and CF. Results from the REF set were added for comparison purposes. For simulation sets ANISO- and ANISO+, results from simulation H of the healthy case are identical to those of the REF set. The loss in pump function after CMI is comparable to that in the REF set, but this loss reduces slightly with increasing anisotropy ratio. For both variations in anisotropy, the increase in work due to patch implantation is higher in P30 than in P120, similar to REF. The effect of the patch decreases with increasing anisotropy ratio, from simulation ANISO- through simulation REF to simulation ANISO+. For set CF, improvement in pump function due to the patch is also higher in P30 than in P120, but the difference is less pronounced than in the other cases. For all sets, the improvement in pump function mainly originates from the native myocardium.

**Fig. 5:**
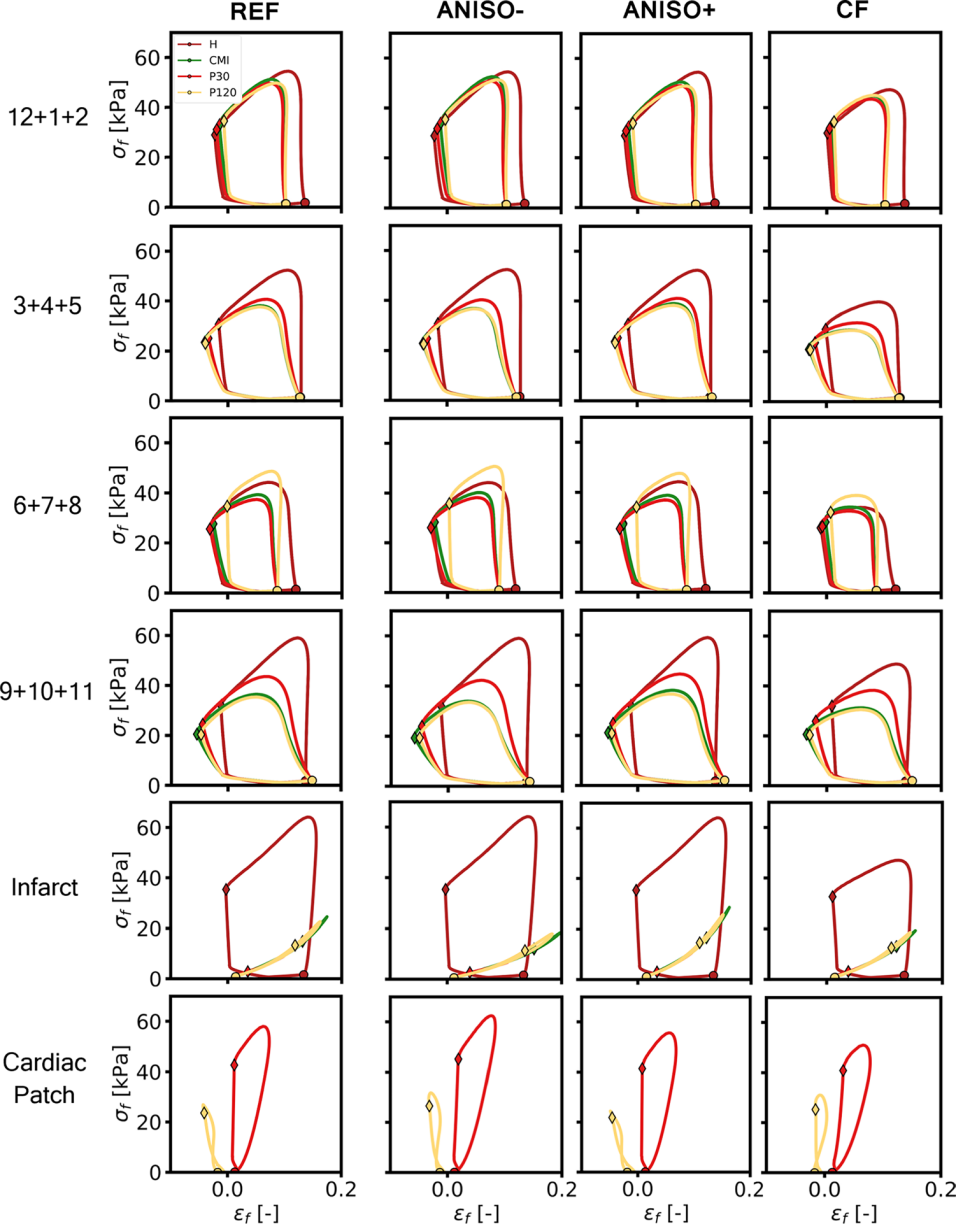
Fiber stress vs. fiber strain, averaged across four sectors in tissue neighboring the infarction and border zone, within the infarct region and within the cardiac patch for simulation sets: REF, ANISO-, ANISO+ and CF

Figure 5 shows the fiber stress vs. strain loops for four sectors neighboring the infarct region. Results from three subsectors are combined as shown in Figure 3a. The simulations of set REF, correspond to Figure 3a. In comparison to simulation set REF, changing the degree of anisotropy in simulations ANISO- and ANISO+ has virtually no effect on the CMI stress–strain loops except for a minor change in isovolumic shortening in sectors 3+4+5 and 9+10+11. Likewise, the effect of adding a patch in simulations P30 and P120 is similar in sets REF, ANISO- and ANISO+.

The effect of variation in the degree of anisotropy is more apparent in the infarct region, where the slope of the stress–strain relation increases with increasing anisotropy ratio. This is in line with the change in anisotropy ratio through a change in tissue stiffness along the fiber direction. In the patch, peak fiber stress decreases with increasing anisotropy ratio.

In set CF, peak fiber stress decreases, local work density is reduced, end-diastolic strain remains constant and end-systolic strain increases in all sectors, comparing the healthy cases H. However, the changes in local fiber mechanics induced by the infarct or subsequent addition of a cardiac patch are similar to those outlined for other simulation sets.

## Discussion

In this study, the restoration of cardiac function was evaluated following the implantation of a contractile cardiac patch over a chronically remodeled infarct. We focused on the effect of the direction of the fibers in the patch. Preliminary findings were reported in (Janssens et al. 2023a). Here, we extended that study by including a border zone neighboring the infarct region, testing additional orientations of the patch and performing a more comprehensive analysis of the results. In addition, we investigated how model outcome depended on the choice of material properties for the healthy and infarct tissue.

### Hemodynamics

The chronically remodeled infarct, 15% in size, was found to cause a 34% loss of pump function. The amount of functional improvement following patch implantation over the chronically infarcted heart was dependent on the orientation of the patch. Our results indicate that the native myocardium contributes about two to three times more to the improvement of pump function than the newly added tissue in the patch (see Figure 2b, right lower chart).

In absolute values, however, these improvements in cardiac function reach up to only 9% of the initial loss found in CMI in the most optimal patch scenario P30. This limited effect is partly due to the thickness of the patch, which was set to 2 mm considering limitations in tissue engineering. Consequently, less than 5 cm$^3$ of contractile tissue was reintroduced to the infarcted heart, where roughly 20 cm$^3$ was lost due to the infarction. The fact that reintroducing 25% of lost tissue does not lead to a 25% recovery of the total loss in cardiac function indicates that other factors besides the amount of tissue determine the effect of the patch on pump function.

### Local tissue function

In the healthy heart, stress–strain loops are similar for all 12 sectors near the infarcted region (see Figure 3a). This is due to the use of an axisymmetric geometry and an optimized orientation of myofibers causing active stress to be transmitted through active myofibers predominantly (Bovendeerd et al. 2009). As explained in our previous study, the disproportional loss in pump function in CMI is related to unfavorable mechanical interactions between the healthy and infarcted tissue (Janssens et al. 2023). The infarct region modeled in that study was a more apical teardrop shape that is typically associated with an occlusion of the left anterior descending (LAD) artery. The LAD infarct affects parts of the LV wall, the septum and the RV wall, and is not well accessible for treatment with a patch. Therefore, infarct location was changed in this study. However, the effects of an LAD and an LCx infarct are both determined by the same principles and are largely explained by the concept of the serial and parallel border zone. In brief, some fibers are in a series arrangement with the infarction and transmit this active stress to the infarcted tissue. This tissue lacks any contractile properties and the stress required to achieve mechanical equilibrium can only be obtained by stretching the infarct. Consequently, these serial fibers in the healthy tissue shorten early as they start to develop active stress during isovolumic contraction. Moreover, the maximum amount of stress that can be generated by these fibers is also reduced following the Frank-Starling principle. In sectors oriented at $90^ \circ$ with respect to sectors with serial fibers, fibers are oriented in parallel to the infarction. Here, mechanical tethering of fibers to the relatively stiff infarcted tissue limits end-diastolic fiber strain. Subsequently, the maximum amount of stress that can be generated is limited as well according to the Frank-Starling principle.

Among the different patch orientations, the largest increase in cardiac function was found in simulation P30. Since, the amount of contractile tissue within the patch remains equal between simulations, these differences can only be attributed to mechanical interactions with the native myocardial tissue. In simulation P30, fibers in the patch are oriented slightly more circumferentially than the epicardial fibers in the healthy myocardium, which are oriented at $45^ \circ$ with respect to the circumferential direction. Consequently, the active fibers in the patch are in series with the fibers in the subepicardial layers of the native myocardium and they counteract the adverse effect that compliant infarcted tissue has on the function of the native fibers. They contribute to the transmission of force, generated by native fibers on top of force transmission through the infarct. This mechanism counteracts the early shortening of fibers observed in CMI and contributes to higher overall cardiac function. Though the fibers in the patch in simulation P60 are oriented fairly well along the epicardial fibers, cardiac function is lower compared to P30. This is likely caused by the transmural variation of the helix angle which decreases from epi- to endocardium. Therefore, overall alignment is better in P30 when taking into account the subepicardial tissue as well.

The mechanical interactions between the patch and native tissue also have implications for tissue functionality within the patch itself. Similar to fibers in the LV that are arranged in parallel with the infarct, mechanical tethering of the cardiac patch to the relatively stiff infarction underneath contributes to low end-diastolic fiber strain. Though this may explain the general lack of mechanical work of fibers within the cardiac patch as compared to that of fibers in the healthy LV, it does not account for the large differences between patch orientations. In all patch simulations, end-diastolic fiber strain in the patch is limited by the tethering effect, causing a decrease in stress generation following the Frank-Starling principle. In P0, P30 and P60, the patch is oriented roughly along the direction of the (sub-)epicardial fibers. These epicardial fibers stretch the infarcted tissue and patch during isovolumic contraction. This stretching augments mechanical work within the patch but operates optimally when epicardial fibers and patch are in alignment. In contrast, in simulations P90, P120 and P150 the patch exerts force orthogonal to the fiber direction. Because of the absence of active fiber stress in the native tissue in this direction, the tissue is relatively compliant which leads to early shortening of fibers and decreased stress generation.

Overall, the cardiac patch contributes to cardiac function via two mechanisms: the direct contribution of the tissue in the patch, and the increased contribution of the native tissue, due to a favorable change in mechanical boundary conditions. Typically, about a third of the total recovery can be attributed to the patch, while the remainder results from restored functionality in native myocardium adjacent to the infarct.

### Parameter variations

In simulations set REF, infarct stiffness is scaled by an isotropic factor $f_\text{pas}$, but this approach does not allow for variations in the ratios between different constituents of the material law. Consequently, the degree of anisotropy in the infarct tissue remains consistent with that of healthy tissue. The anisotropy change in simulation sets ANISO- and ANISO+ induces a systematic change in local fiber mechanics while the effects of the infarction or patch implantation persist (see Figure 5). A reduction in anisotropy reduces infarct stiffness along the fiber direction, leading to more isovolumic shortening in series-arranged fibers and a higher strain excursion for fibers in the infarct itself. Fibers in the cardiac patch are stretched slightly further during isovolumic contraction and hence present with higher peak fiber stress following the Frank-Starling principle. Increased anisotropy shows exactly the opposite trends because of an increase in stiffness along the fiber direction.

The introduction of 25% active stress in cross-fiber direction reduces cardiac pump function in the healthy heart (see Figure 4). This reduction is related to the development of active stress in transmural direction, that opposes the thickening of the wall that naturally occurs during ejection. The reduction in cardiac stroke work is reflected in a reduction of tissue work density, apparent in the reduction of the area enclosed by the stress–strain loops (see Figure 5). The changes in stress–strain loops induced by the infarct and cardiac patch are similar compared to simulation set REF. The infarct induces isovolumic shortening in series-arranged fibers and restricts end-diastolic strain and peak fiber stress in parallel fibers through mechanical tethering. Similar to set REF, the addition of a cardiac patch reduces isovolumic shortening and increases peak fiber stress in the fibers that lie in line with the patch (see Figure 5).

### Study limitations

In this study, we chose to model myocardial infarction through the local absence of active stress development and an increase in passive tissue stiffness. We did not include the thinning of the ventricular wall, which is reported to reduce to about 60% of its initial thickness (Richardson et al. 2015). In our previous study, we observed that a reduction in infarct thickness primarily increases strain at end-diastole. It hardly affects strain at end-ejection but it does increase maximum stress in the parallel border zone (Janssens et al. 2023). An increase in infarct tissue stiffness primarily reduces strain at end-diastole in the parallel border zone, but not in the serial border zone. It hardly affects strain at end-ejection and tends to decrease maximum stress. We expect the changes induced by a variation in infarct thickness or stiffness to affect patch functionality through the mechanical tethering effect. Extending our model with these variations may provide a more detailed analysis and valuable insights.

Furthermore, it has been reported that the orientation of collagen fibers in the infarct region and border zone in healing scar tissue is typically different from that of healthy myocardium (French and Holmes 2019). Collagen fibers in the infarct region can adopt a highly aligned or totally random distribution of fibers which is thought to arise from local stretch patterns in the infarct region during the healing process. Animal models in which the infarct tissue is stretched isotropically develop random fiber distributions, while animal models in which stretch is uniaxial develop aligned collagen fibers (Richardson et al. 2015). In this respect, infarct location, size and transmurality can also be of importance as these influence the stretching pattern of the infarct post-MI. Still, modeling the infarct as an isotropic tissue is equivalent to modeling a random collagen fiber orientation at tissue level. Therefore, considering the results of ANISO- and the relatively small degree of anisotropy in passive tissue compared to active properties, we do not anticipate large differences.

We also assumed simultaneous activation of myofibers in both the LV and cardiac patch. While incorporating asynchronous fiber activation is feasible, we opted for simultaneous activation as previous studies indicated more physiological strain patterns were obtained with synchronous activation in our model (Kerckhoffs et al. 2003). The findings of that study suggested myocardial tissue may be able to synchronize the onset of contraction despite asynchrony of depolarization through an electromechanical delay. Nevertheless, some studies already indicate fiber activation in the cardiac patch is likely delayed compared to the host myocardium due to the lower conductivity that is typically associated with cardiac patches (Tenreiro et al. 2021; Rosales et al. 2024). Further results should point out how delayed electrical activation affects the mechanical activation of fibers in the cardiac patch and the consequences in terms of cardiac function.

The effect of the patch, observed in our model, depends on the realism of the model for the chronic infarct. Comparison of predictions on stress and strain with experimental data is challenging since local stress cannot be measured. While, strain can be measured, often only global strain measures or averaged strain values over a wall segment are reported as in Bachner-Hinenzon et al. (2016).

In our previous study (Janssens et al. 2023), we compared findings from our model to those from other modeling studies. In particular, the model of Leong et al. (2017) provides a good comparison as it also employs a model of chronic MI with a three-fold increase in infarct stiffness in a truncated ellipsoidal geometry. The results are very similar: end-systolic fiber stress in the border and adjacent remote zones is decreased when fibers are oriented perpendicular to the infarct boundary and elevated when fibers are oriented tangential to this boundary. The finding that stress elevation is lower in our model can be partly explained by the LV afterload. In Leong et al. (2017), the pressure at aortic valve opening is fixed, whereas in our closed circulation model, it is reduced due to the reduced pumping performance of the infarcted LV. This reduction of about 14% in pressure translates into an overall reduction of stress levels by about the same amount in the infarcted LV compared to the healthy heart.

In this study, we assumed that the material properties of the cardiac patch were equal to those of the native healthy myocardium. This assumption was an initial step in capturing cardiac patch mechanics, guided by the logical aim to achieve properties similar to native tissue. However, it is important to mention that passive material properties of the patch are dependent on the scaffolds’ properties and collagen formation. Furthermore, the cells in engineered heart tissues typically exhibit significantly lower force generation compared to native myocardium (Tzatzalos et al. 2016). The current lower contractile capacity could potentially limit the functional improvements described in this study.

We also did not take into account the right ventricle (RV). Adding the RV may be expected to change local stress and strain patterns near the RV attachment sites. We expect effects in the LV free wall, where we positioned the infarcted region, to be small. In addition, we expect that the mechanisms that we identified to play a role in the local function of cardiac and patch tissue would not be affected by the RV.

Mesh convergence was verified by changing the mesh resolution to both a coarser and a finer mesh. These consisted of 47,177 and 92,487 elements, respectively. Between these resolutions, hemodynamic markers SV, EF, $W_\text{hemo}$ varied no more than 0.5%. Changes in average fiber stress and strain in the cardiac patch, infarct region, border zone and the remote tissue at end-diastole, mid-systole and end-systole remained within 0.005 and 3 kPa, respectively.

### Clinical relevance

Even in the most favorable situation P30, only 9% of the cardiac function lost through MI was recovered. For clinical applications, the risk associated with surgery would outweigh a low potential increase in cardiac function and render this treatment non-beneficial. However, if developments in tissue engineering would allow for the creation of patches with a thickness well above the 2 mm thickness that we used in our study, the results of our analysis on patch orientation would become more relevant clinically.

Mechanical tethering of the patch to the infarct also limits treatment efficacy through restricted end-diastolic fiber strain combined with the Frank-Starling effect. Therefore, in cases of lower infarct stiffness or left ventricular aneurysm, a higher degree of functional restoration might be obtained.

While a direct improvement of pump function is desirable, cardiac patches can also be considered successful if the progression of heart failure is stalled. Our study suggests the patch is capable of restoring the local mechanical environment in series-arranged fibers which might prevent adverse ventricular remodeling. Further research should focus on suitable time-points for patch implantation during the remodeling process, whether certain patient subsets may benefit more from treatment or what the long-term consequences in terms of growth and remodeling are. Still, our analysis suggests that the orientation of the patch would be an important determinant to consider, given that the amount of cardiac function restored is equal to 9% in P30 compared to only 3% in P90.

## Figures and Tables

**Fig. 6:**
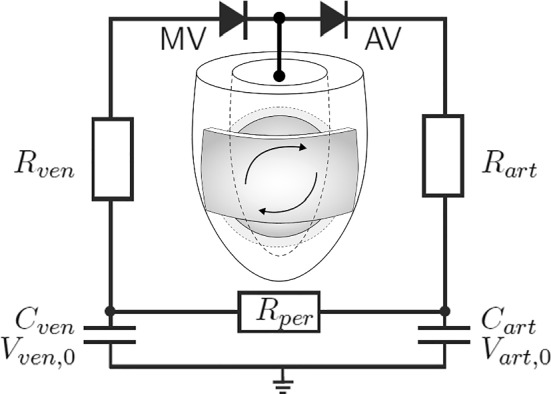
Lumped parameter model of the circulation. MV, mitral valve; AV, aortic valve; $R_\text{ven}$, venous resistance; $R_\text{art}$, characteristic arterial impedance; $R_\text{per}$, peripheral resistance; $C_\text{ven}$, venous compliance; $C_\text{art}$, arterial compliance; $V_\text{ven,0}$, zero pressure venous volume; $V_\text{art,0}$, zero pressure arterial volume

## Data Availability

The data that support the findings of this study are available from the corresponding author upon reasonable request.

## FE model of LV mechanics in MI supported by a cardiac patch

**Geometry** A thick-walled, truncated, confocal ellipsoid approximates the LV geometry in its unstressed state. Cavity and wall volumes were set to 44 and 136 ml, respectively. A prolate ellipsoidal coordinate system was used to define the LV geometry and relates to the Cartesian system, with coordinates (*x*, *y*, *z*), according to:

4 $$\begin{aligned} \begin{array}{ll} x = C \sinh (\xi ) \sin (\theta ) \cos (\phi )\\ y = C \sinh (\xi ) \sin (\theta ) \sin (\phi )\\ z = C \cosh (\xi ) \cos (\theta ) \end{array} \end{aligned}$$

where C is the focal length of 4.3 cm and $\xi$, $\theta$ and $\phi$ represent the radial, longitudinal and circumferential coordinates, respectively. In ellipsoidal coordinates, the LV geometry is described by $\xi _\text{endo} \le \xi \le \xi _\text{epi}$, $\theta _\text{base} \le \theta \le \pi$ and $0 \le \phi \le 2\pi$, where $\xi _\text{ endo } = 0.37$, $\xi _\text{ epi } = 0.68$ and $\theta _\text{base}=\theta (z_\text{base}=24mm)$. An additional layer of elements, measuring 2 mm in thickness, was added onto the outer wall of the ellipsoid to model the cardiac patch. The boundary between the epicardium and cardiac patch was preserved during the meshing procedure.

The definitions of the infarct region, border zone and cardiac patch are described in the main text.

**Fiber orientation** A rule-based method was used to define the fiber orientation in terms of a helix angle ($\alpha _h$) and transverse angle ($\alpha _t$). Normalized legendre polynomials were used to describe the nonlinear endo-to-epicardial and base-to-apex variation of $\alpha _h$ and $\alpha _t$. The polynomials were defined on normalized wall-bound coordinates *u* and *v*. The longitudinal coordinate *u*, varies linearly with the distance from the equatorial plane, as measured along the meridional curve through the points of interest, from $u= +0.5$ in the basal plane through $u = 0$ at the equator to $u = -1$ at the apex. The transmural coordinate *v* varies linearly with the actual distance in the ventricular wall, from $v = -1$ at the endocardial surface to $v = +1$ at the epicardial surface. The helix and transverse angels are then given by:

5 $$\begin{aligned} \alpha _h(u, v) =&\,[h_{10}L_0\,(v) + h_{11}L_1\,(v)\nonumber \\ + h_{12}&L_2\,(v) + h_{13}L_3\,(v) + h_{14}L_4\,(v)]\nonumber \\&\qquad \quad \cdot [1 + h_{22}L_2\,(u) + h_{24}L_4\,(u)] \end{aligned}$$

6 $$\begin{aligned} \alpha _t(u, v)&\nonumber \\ = [1&+ t_{11}L_1\,(v) + t_{12}L_2\,(v)]\cdot (1-v^2) \nonumber \\&\qquad \cdot [t_{21}L_1\,(u) + t_{23}L_3\,(u) + t_{25}L_5\,(u)] \end{aligned}$$

where $h_{22}$, $h_{24}$, $t_{11}$ and $t_{12}$ are dimensionless parameters and $h_{10}$, $h_{11}$, $h_{12}$, $h_{13}$, $h_{14}$, $t_{21}$, $t_{23}$ and $t_{25}$ are expressed in radians. The polynomials $L_i(v)$ and $L_i(u)$ are normalized Legendre polynomials of order i. Values of the fiber angle parameters are shown in Table 1.

**Material properties** Passive material behavior $\varvec{\sigma }_{pas}$ is related to the strain energy density function (*W*) according to:

7 $$\begin{aligned}{} & {} \varvec{\sigma }_\text{pas} = \dfrac{1}{\det (\varvec{F})}\varvec{F}\cdot \dfrac{\partial W}{\partial \varvec{E}}\cdot \varvec{F}^T \end{aligned}$$

8 $$\begin{aligned}{} & {} \varvec{E} = \dfrac{1}{2}(\varvec{F}^T\cdot \varvec{F}-\varvec{I}) \end{aligned}$$

with $\varvec{F}$ the deformation gradient tensor, $\varvec{E}$ the Green-Lagrange strain tensor, and $\varvec{I}$ the unity tensor. The strain energy density function *W* is composed of a term describing the response to a change in shape ($W_s$) and a term representing near-incompressibility ($W_v$):

9 $$\begin{aligned} W = W_s + W_v \end{aligned}$$

Formulated in terms of components of the Green-Lagrange strain tensor $\varvec{E}$ with respect to the material-bound coordinates system $[\varvec{\vec {e}}_f, \varvec{\vec {e}}_n, \varvec{\vec {e}}_s]$, $W_s$ reads as follows:

10 $$\begin{aligned}{} {} W_\text{ s } = a_0[\text{ exp}(Q) - 1] \end{aligned}$$

11 $$\begin{aligned} Q &= a_1 (E^2_\text{ ff }+E^2_\text{ ss }+E^2_\text{ nn}) + a_3 E^2_\text{ ff } + \dfrac{a_2}{2} (E^2_{ns}\nonumber \\ & \,\,+ E^2_\text{ sn } + E^2_\text{ fs } + E^2_\text{ sf } + E^2_\text{ fn } + E^2_\text{ nf}) \end{aligned}$$

The volumetric term reads:

12 $$\begin{aligned} W_v = a_5[\det (\varvec{F}^T\cdot \varvec{F})-1]^2 \end{aligned}$$

Active material behavior $\sigma _\text{act}$ is modeled through a series arrangement of a contractile element and a series elastic element. The magnitude of $\sigma _\text{act}$ depends on the time elapsed since activation ($t_\text{a}$), sarcomere length ($l_\text{s}$), and contractile element length ($l_\text{c}$) according to:

13 $$\begin{aligned} \sigma _\text{act} = \dfrac{l_\text{s}}{l_{s0}}f_{\text{iso}}\,(l_c) f_\text{twitch}\,(t_a, l_\text{s})E_a\,(l_\text{s}-l_\text{c}) \end{aligned}$$

where $l_{\text{s}0}$ is the sarcomere length in the stress-free state and $E_a$ is the stiffness of the series elastic element. The length ($f_\text{iso}$) and time dependence ($f_\text{twitch}$) are modeled as follows:

14 $$\begin{aligned}{} & {} f_\text{iso}\,(l_\text{c}) = \Bigg \{\begin{array}{lll} &{}T_0 \; \text {tanh}^2\,[a_l(l_\text{c}-l_{\text{c},\,0})] &{}l_\text{c} \ge l_{\text{c},\,0} \\ {} &{}0 &{}l_\text{c} < l_{c,\,0}\end{array} \end{aligned}$$

15 $$\begin{aligned}{} & {} \begin{aligned}&f_\text{ twitch }(l_c)  =\,\left\{ \begin{array}{ll} 0 &{} 0 > t_\text{ a }\\ \text {tanh}^2 \Big (\dfrac{t_\text{ a }}{\tau _r}\Big ) \text {tanh}^2 \Big (\dfrac{t_{m}-t_a}{\tau _d}\Big ) &{} 0 \le t_\text{ a } \le t_\text{ m }\\ 0 &{} t_a < t_{m} \end{array}\right. \end{aligned} \end{aligned}$$

16 $$\begin{aligned}{} {} t_{m} = b(l_\text{s}-l_d) \end{aligned}$$

where $T_0$ is the reference active stress level, $a_t$ governs the steepness of the stress-length curve, $l_{\text{c, }0}$ is the contractile element length below which active stress is zero, $\tau _r$ and $\tau _d$ govern the rise and decay time of the twitch, $t_\text{max}$ is twitch duration, *b* describes the increase of twitch duration with sarcomere length, and $l_d$ is sarcomere length, where twitch duration is zero. The time course of $l_\text{c}$ is described by the following:

17 $$\begin{aligned} \dfrac{\text {d}l_c}{\text {d}t} = [E_a(l_\text{s}-l_\text{c})-1]v_0 \end{aligned}$$

, where $v_0$ is the unloaded shortening velocity. Development of $\sigma _{act}$ was initiated simultaneously throughout the LV wall with a cardiac cycle time of 800 ms.

**Governing equations and boundary conditions** In the model, the quasistatic equations of conservations of linear momentum are solved.

18 $$\begin{aligned} \vec {\nabla } \cdot \varvec{\sigma } = \vec {0} \end{aligned}$$

At the base, rigid body motion was suppressed using Dirichlet boundary conditions. For all nodes in the basal plane, out-of-plane displacement was suppressed and in-plane displacement was confined by restricting the solution of this subset of nodes to its null space. Neumann boundary conditions were used to subject the endocardial surface to a uniform LV pressure $p_{\text{lv}}$. During the isovolumetric phases, $p_{\text{lv}}$ is determined by the mechanical equilibrium with the myocardial tissue at constant end-diastolic or end-systolic volume. During the filling and ejection phase, $p_{\text{lv}}$ is governed by a 0D closed-loop lumped parameter model of the systemic circulatory system (see Figure 6). In this model, the aortic and mitral valves are modeled as ideal diodes. Arteries, veins and peripherals are represented through constant resistances (*R*) and capacitances (*C*). The pressure drop ($\Delta$p) across each of these components is given by:

19 $$\begin{aligned}{} {} \Delta p_\text{c} = \dfrac{V-V_0}{C} \end{aligned}$$

20 $$\begin{aligned}{} {} \Delta p_\text{R} = \text{ Rq } \end{aligned}$$

where *V* is the volume in the capacitance and q is the flow through the resistance. The pressure-volume relation of the capacitance represents a linearization around the physiological working point, where $V_0$ is the volume at zero pressure. The sum of the blood volumes in the LV cavity and the arterial and venous capacitances is set equal to the total blood volume ($V_\text{blood}$).

**Table 1:** Parameter values used in the FE model for the fiber orientation, passive material properties, active material properties and circulation model. $^*$ The non-zero value of $\beta$ only holds for the CF set of simulations

Parameters	Value
Fiber angles	
$h_{10}$, rad	0.362
$h_{11}$, rad	$-$1.16
$h_{12}$, rad	$-$0.124
$h_{13}$, rad	0.129
$h_{14}$, rad	$-$0.0614
$h_{22}$	0.0984
$h_{24}$	$-$0.0701
$t_{11}$	$-$0.626
$t_{12}$	0.502
$t_{21}$,rad	0.626
$t_{23}$, rad	0.211
$t_{25}$, rad	0.038
Passive material	
$a_0$, kPa	0.4
$a_1$	3.0
$a_2$	6.0
$a_3$	3.0
$a_5$, kPa	55.0
Active material	
$T_0$, kPa	160
$E_a$, $\mu$ m$^{-1}$	20
$a_l$, $\mu$ m$^{-1}$	2
$l_{c0}$, $\mu$m	1.5
$l_{s0}$, $\mu$m	1.9
$\tau _r$, ms	75
$\tau _d$, ms	150
*b*, ms/$\mu$m	160
$l_d$, $\mu$m	$-$0.5
$\beta$	0 (0.25)$^*$
Circulation	
$R_{art}$, kPa$\cdot$s$\cdot$ml$^{-1}$	0.010
$R_{per}$, kPa$\cdot$s$\cdot$ml$^{-1}$	0.12
$R_{ven}$, kPa$\cdot$s$\cdot$ml$^{-1}$	0.005
$C_\text {art}$, ml/kPa	25
$C_\text {ven}$, ml/kPa	600
$V_\text {art. 0}$, ml	500
$V_\text {ven, 0}$, ml	3000
$V_\text {blood}$, ml	5000
